# Supplementary material for: Selected Alu methylation levels in the gastric carcinogenesis cascade
Source: PeerJ. 2025 May 20;13:e19485. doi: 10.7717/peerj.19485 (PMC12101442; doi:10.7717/peerj.19485)
Supplement: Supplemental Information 2 [file peerj-13-19485-s002.docx]

Table SI Detailed data of Alu methylation levels in tissue involving in gastric carcinogenesis cascade

| **Code** | **Sex** | **Age** | ***H. Pylori* infection** | **EBV infection** | **% Alu methylation** | | | | |
| --- | --- | --- | --- | --- | --- | --- | --- | --- | --- |
|  |  |  |  |  | **%mC** | **%mCmC** | **%uCmC** | **%mCuC** | **%uCuC** |
| Normal stomach | | | | | | | | | |
| NG01 | Female | 62 | Negative | Negative | 38.82 | 9.60 | 32.89 | 25.61 | 32.16 |
| NG02 | Female | 50 | Positive | Negative | 41.06 | 13.52 | 32.80 | 24.58 | 30.70 |
| NG03 | Male | 84 | Negative | Negative | 43.80 | 11.67 | 32.22 | 25.22 | 31.83 |
| NG04 | Male | 93 | Negative | Negative | 40.39 | 12.37 | 32.57 | 25.12 | 31.14 |
| NG05 | Female | 40 | Negative | Negative | 41.02 | 12.14 | 33.56 | 25.59 | 29.87 |
| NG06 | Female | 18 | Negative | Negative | 39.02 | 11.31 | 33.13 | 23.77 | 33.16 |
| NG07 | Female | 74 | Negative | Negative | 44.25 | 18.00 | 33.60 | 24.79 | 28.54 |
| NG08 | Female | 23 | Negative | Negative | 46.96 | 37.42 | 29.07 | 23.92 | 28.50 |
| NG09 | Male | 45 | Negative | Negative | 43.16 | 15.60 | 34.31 | 24.95 | 29.00 |
| NG10 | Female | 90 | Negative | Negative | 42.79 | 15.34 | 33.81 | 25.08 | 29.43 |
| NG11 | Male | 50 | Negative | Negative | 50.12 | 27.49 | 34.12 | 23.64 | 24.34 |
| NG12 | Female | 73 | Negative | Negative | 47.47 | 29.64 | 22.60 | 27.48 | 29.46 |
| NG13 | Male | 52 | Negative | Negative | 44.88 | 18.91 | 33.14 | 25.27 | 28.04 |
| NG14 | Male | 55 | Negative | Negative | 45.34 | 20.93 | 32.33 | 24.51 | 28.35 |
| Chronic gastritis | | | | | | | | | |
| CG01 | Male | 80 | Negative | Negative | 42.14 | 15.90 | 30.23 | 25.13 | 31.04 |
| CG02 | Female | 56 | Positive | Negative | 40.64 | 15.27 | 29.08 | 24.42 | 33.12 |
| CG03 | Female | 56 | Positive | Negative | 44.41 | 20.59 | 29.33 | 23.29 | 30.35 |
| CG04 | Female | 31 | Positive | Negative | 46.03 | 21.81 | 29.17 | 24.73 | 28.45 |
| CG05 | Female | 58 | Positive | Negative | 44.88 | 18.06 | 32.52 | 24.72 | 27.89 |
| CG06 | Male | 63 | Negative | Negative | 46.79 | 23.77 | 30.98 | 21.31 | 28.48 |
| CG07 | Male | 64 | Negative | Negative | 47.86 | 23.61 | 30.74 | 23.86 | 26.59 |
| CG08 | Female | 56 | Negative | Negative | 49.99 | 27.29 | 30.70 | 22.42 | 25.45 |
| CG09 | Female | 68 | Positive | Negative | 46.15 | 21.03 | 32.32 | 22.85 | 27.73 |
| CG10 | Female | 40 | Positive | Negative | 41.19 | 16.43 | 28.40 | 24.41 | 32.97 |
| CG11 | Female | 48 | Positive | Negative | 45.15 | 18.55 | 31.21 | 25.87 | 27.76 |
| CG12 | Female | 54 | Positive | Negative | 43.94 | 16.51 | 31.32 | 26.51 | 28.44 |
| CG13 | Male | 61 | Negative | Negative | 46.34 | 13.56 | 33.49 | 25.89 | 31.39 |
| CG14 | Male | 65 | Positive | Negative | 45.60 | 12.53 | 32.05 | 27.32 | 31.95 |
| CG15 | Male | 68 | Positive | Negative | 45.79 | 14.52 | 31.08 | 26.44 | 32.63 |
| CG16 | Female | 68 | Negative | Negative | 45.23 | 13.25 | 31.37 | 26.62 | 32.82 |
| CG17 | Female | 73 | Negative | Negative | 45.39 | 14.58 | 30.44 | 26.40 | 33.22 |
| CG18 | Male | 81 | Negative | Negative | 45.55 | 14.48 | 30.96 | 26.24 | 32.95 |
| CG19 | Male | 36 | Negative | Negative | 45.16 | 14.65 | 30.18 | 26.21 | 33.60 |
| CG20 | Male | 68 | Negative | Negative | 46.22 | 14.84 | 31.19 | 26.68 | 32.17 |
| CG21 | Female | 65 | Positive | Negative | 46.33 | 16.63 | 30.08 | 26.04 | 32.88 |
| CG22 | Male | 69 | Negative | Negative | 45.80 | 17.32 | 29.30 | 25.24 | 33.96 |
| CG23 | Male | 70 | Positive | Negative | 42.58 | 14.17 | 28.09 | 24.71 | 37.03 |
| CG24 | Male | 31 | Positive | Negative | 43.93 | 14.16 | 35.53 | 26.30 | 25.94 |
| CG25 | Male | 64 | Negative | Negative | 46.94 | 20.93 | 33.03 | 25.22 | 24.97 |
| CG26 | Female | 73 | Negative | Negative | 43.73 | 16.65 | 32.12 | 25.93 | 27.94 |
| CG27 | Female | 56 | Negative | Negative | 42.62 | 15.44 | 32.60 | 25.07 | 29.15 |
| CG28 | Female | 69 | Negative | Negative | 38.08 | 12.95 | 33.02 | 19.79 | 35.69 |
| CG29 | Female | 56 | Negative | Negative | 38.71 | 10.38 | 31.98 | 25.27 | 32.87 |
| CG30 | Male | 76 | Negative | Negative | 45.51 | 36.25 | 26.97 | 24.37 | 30.45 |
| CG31 | Male | 75 | Negative | Negative | 44.24 | 27.50 | 31.37 | 23.88 | 29.59 |
| CG32 | Female | 63 | Negative | Negative | 43.62 | 32.94 | 27.86 | 22.61 | 32.36 |
| Intestinal metaplasia | | | | | | | | | |
| IM01 | Male | 41 | Negative | Negative | 37.86 | 9.42 | 34.54 | 22.24 | 33.67 |
| IM02 | Female | 22 | Negative | Negative | 36.98 | 8.26 | 33.50 | 24.28 | 34.10 |
| IM03 | Female | 72 | Negative | Negative | 37.36 | 7.66 | 33.82 | 26.22 | 32.56 |
| IM04 | Male | 56 | Negative | Negative | 36.89 | 7.27 | 35.02 | 24.95 | 33.10 |
| IM05 | Female | 67 | Positive | Negative | 38.37 | 9.35 | 31.94 | 26.12 | 32.51 |
| IM06 | Male | 85 | Negative | Negative | 38.65 | 9.94 | 34.34 | 22.86 | 32.62 |
| IM07 | Female | 39 | Negative | Negative | 39.20 | 9.47 | 33.82 | 25.71 | 30.88 |
| IM08 | Female | 49 | Negative | Negative | 38.20 | 8.22 | 37.82 | 22.59 | 31.47 |
| IM09 | Female | 49 | Negative | Negative | 39.53 | 10.83 | 32.84 | 24.08 | 31.83 |
| IM10 | Male | 61 | Positive | Negative | 36.53 | 6.98 | 34.50 | 25.43 | 33.51 |
| IM11 | Male | 55 | Positive | Negative | 36.81 | 8.23 | 29.96 | 27.57 | 34.43 |
| IM12 | Male | 52 | Negative | Negative | 37.92 | 8.36 | 32.20 | 27.32 | 32.25 |
| IM13 | Male | 64 | Negative | Negative | 38.76 | 8.44 | 36.72 | 24.64 | 31.08 |
| IM14 | Female | 82 | Positive | Negative | 41.46 | 10.16 | 34.45 | 24.42 | 31.20 |
| IM15 | Male | 54 | Negative | Negative | 39.80 | 10.22 | 36.52 | 25.84 | 28.18 |
| IM16 | Female | 60 | Negative | Negative | 38.91 | 8.92 | 34.37 | 26.08 | 31.37 |
| IM17 | Female | 57 | Positive | Negative | 38.65 | 9.09 | 34.10 | 25.28 | 32.06 |
| IM18 | Female | 67 | Positive | Negative | 40.89 | 11.45 | 32.32 | 25.41 | 30.70 |
| IM19 | Male | 70 | Negative | Negative | 40.69 | 9.85 | 34.48 | 27.32 | 29.18 |
| IM20 | Female | 66 | Negative | Negative | 38.77 | 8.17 | 33.57 | 28.78 | 30.75 |
| IM21 | Female | 76 | Negative | Negative | 39.11 | 8.82 | 33.89 | 27.34 | 30.88 |
| IM22 | Male | 63 | Negative | Negative | 38.18 | 8.13 | 32.95 | 28.24 | 31.78 |
| IM23 | Female | 51 | Positive | Negative | 39.30 | 10.13 | 31.51 | 26.47 | 32.09 |
| IM24 | Male | 70 | Positive | Negative | 39.26 | 9.10 | 34.81 | 25.89 | 30.93 |
| IM25 | Female | 44 | Negative | Negative | 37.13 | 11.78 | 28.67 | 20.25 | 39.38 |
| IM26 | Female | 76 | Positive | Negative | 36.13 | 10.64 | 29.93 | 19.91 | 40.19 |
| IM27 | Male | 69 | Negative | Negative | 35.68 | 10.29 | 30.80 | 18.99 | 40.87 |
| IM28 | Female | 30 | Positive | Negative | 37.70 | 9.88 | 34.17 | 21.10 | 34.42 |
| IM29 | Male | 78 | Negative | Negative | 39.42 | 12.25 | 32.92 | 19.72 | 33.71 |
| IM30 | Female | 83 | Negative | Negative | 38.44 | 10.84 | 34.09 | 20.20 | 34.02 |
| IM31 | Female | 71 | Negative | Negative | 40.53 | 12.89 | 32.14 | 21.21 | 31.66 |
| IM32 | Female | 79 | Negative | Negative | 42.70 | 13.11 | 31.48 | 25.87 | 27.36 |
| IM33 | Female | 60 | Negative | Negative | 42.78 | 12.52 | 36.33 | 22.71 | 26.28 |
| IM34 | Male | 41 | Negative | Negative | 43.73 | 13.82 | 35.03 | 22.58 | 25.93 |
| IM35 | Female | 54 | Negative | Negative | 43.03 | 13.31 | 34.58 | 22.87 | 26.86 |
| IM36 | Male | 85 | Negative | Negative | 41.81 | 12.08 | 33.91 | 24.24 | 27.97 |
| Dysplasia | | | | | | | | | |
| DL01 | Female | 72 | Negative | Negative | 39.59 | 10.51 | 32.14 | 25.48 | 30.95 |
| DL02 | Male | 60 | Negative | Negative | 35.86 | 7.30 | 32.05 | 26.15 | 35.13 |
| DL03 | Female | 73 | Negative | Negative | 35.45 | 6.72 | 33.13 | 25.75 | 35.23 |
| DL04 | Female | 65 | Negative | Negative | 38.95 | 9.43 | 34.27 | 24.86 | 30.87 |
| DL05 | Female | 92 | Negative | Negative | 40.35 | 10.06 | 36.13 | 24.30 | 28.45 |
| DL06 | Male | 70 | Negative | Negative | 40.54 | 11.96 | 31.69 | 24.03 | 30.85 |
| DL07 | Male | 75 | Negative | Negative | 42.71 | 13.89 | 29.46 | 25.79 | 28.52 |
| DL08 | Male | 63 | Negative | Negative | 45.29 | 32.21 | 30.02 | 23.74 | 29.47 |
| DL09 | Male | 49 | Negative | Negative | 49.48 | 47.24 | 29.95 | 20.49 | 27.71 |
| DL10 | Male | 72 | Negative | Negative | 46.39 | 37.43 | 31.02 | 20.99 | 29.42 |
| DL11 | Male | 74 | Negative | Negative | 46.47 | 36.95 | 29.31 | 23.17 | 29.14 |
| DL12 | Female | 55 | Negative | Negative | 45.98 | 35.02 | 30.90 | 22.10 | 29.28 |
| DL13 | Female | 72 | Negative | Negative | 38.35 | 11.06 | 31.12 | 22.39 | 34.64 |
| DL14 | Female | 72 | Negative | Negative | 39.57 | 12.51 | 29.28 | 22.98 | 33.72 |
| DL15 | Female | 73 | Negative | Negative | 39.95 | 11.98 | 31.45 | 23.06 | 31.72 |
| Adenocarcinoma | | | | | | | | | |
| AD01 | Female | 71 | Negative | Negative | 40.83 | 14.56 | 27.72 | 21.77 | 33.75 |
| AD02 | Female | 75 | Negative | Negative | 41.47 | 14.36 | 29.16 | 22.25 | 31.59 |
| AD03 | Male | 70 | Negative | Negative | 39.19 | 17.28 | 30.09 | 21.39 | 35.51 |
| AD04 | Male | 82 | Negative | Negative | 41.44 | 23.30 | 28.99 | 20.33 | 34.21 |
| AD05 | Female | 40 | Positive | Negative | 38.74 | 17.94 | 29.90 | 20.17 | 37.14 |
| AD06 | Male | 69 | Negative | Positive | 40.83 | 19.09 | 32.17 | 20.47 | 32.66 |
| AD07 | Male | 68 | Positive | Negative | 39.64 | 18.83 | 31.67 | 19.20 | 35.50 |
| AD08 | Female | 85 | Positive | Negative | 41.04 | 19.98 | 33.74 | 18.53 | 32.78 |
| AD09 | Female | 59 | Negative | Negative | 40.94 | 20.69 | 31.03 | 20.16 | 33.55 |
| AD10 | Female | 70 | Negative | Negative | 39.45 | 16.74 | 31.55 | 21.01 | 34.45 |
| AD11 | Female | 62 | Negative | Negative | 36.54 | 10.07 | 31.59 | 22.56 | 36.95 |
| AD12 | Male | 70 | Positive | Positive | 37.24 | 11.08 | 35.01 | 19.63 | 35.91 |
| AD13 | Female | 50 | Positive | Negative | 40.51 | 21.77 | 28.92 | 20.31 | 35.46 |
| AD14 | Male | 49 | Positive | Negative | 39.52 | 16.69 | 26.73 | 25.59 | 34.22 |
| AD15 | Female | 60 | Negative | Positive | 39.43 | 11.91 | 30.64 | 23.43 | 32.72 |
| AD16 | Female | 75 | Negative | Negative | 38.53 | 10.58 | 32.08 | 23.20 | 33.11 |
| AD17 | Male | 61 | Negative | Negative | 37.93 | 11.04 | 28.22 | 25.01 | 35.56 |
| AD18 | Female | 77 | Negative | Negative | 41.17 | 12.20 | 33.20 | 23.71 | 28.25 |
| AD19 | Female | 42 | Negative | Negative | 38.82 | 11.52 | 29.91 | 23.89 | 33.81 |
| AD20 | Female | 94 | Negative | Negative | 38.11 | 8.37 | 37.19 | 22.15 | 30.72 |
| AD21 | Male | 51 | Negative | Positive | 39.47 | 12.83 | 29.97 | 21.91 | 34.09 |
| AD22 | Female | 27 | Positive | Negative | 36.63 | 8.84 | 30.99 | 24.62 | 35.68 |
| AD23 | Female | 76 | Positive | Negative | 37.70 | 8.99 | 32.62 | 24.80 | 32.89 |
| AD24 | Female | 78 | Negative | Negative | 38.41 | 10.26 | 31.04 | 24.92 | 32.93 |
| AD25 | Female | 78 | Negative | Negative | 35.91 | 6.89 | 35.39 | 23.05 | 34.55 |
| AD26 | Female | 55 | Negative | Negative | 37.62 | 9.54 | 32.77 | 23.03 | 34.01 |
| AD27 | Female | 64 | Negative | Negative | 35.92 | 6.38 | 32.77 | 32.52 | 31.84 |
| AD28 | Female | 51 | Negative | Negative | 36.28 | 6.80 | 36.34 | 27.74 | 31.82 |
| AD29 | Female | 63 | Negative | Negative | 35.70 | 7.20 | 36.48 | 24.12 | 34.25 |

Table S2 Multivariate regression analysis on 14 Alu-methylated Normal stomach patients.

|  | Adjusted β | 95 % CI | *p*-value |
| --- | --- | --- | --- |
| Pattern of Alu methylation | | | |
| mC  Sex  Age | -2.05  -0.011 | -6.20-2.10  -0.10-0.081 | 0.301  0.795 |
| mCmC  Sex  Age | -0.33  -0.093 | -10.75-10.08  -0.32-0.14 | 0.944  0.394 |
| uCmC  Sex  Age | -1.85  -0.017 | -5.65-1.96  -0.10-0.068 | 0.308  0.662 |
| mCuC  Sex  Age | 0.53  0.023 | -0.52-1.58  -0.00073-0.046 | 0.290  0.056 |
| uCuC  Sex  Age | 1.57  0.014 | -1.12-4.26  -0.046-0.074 | 0.225  0.628 |

Table S3 Multivariate regression analysis on 32 Alu-methylated Chronic gastritis patients.

|  | Adjusted β | 95 % CI | *p*-value |
| --- | --- | --- | --- |
| Pattern of Alu methylation | | | |
| mC  Sex  Age | -1.51  -0.0057 | -3.35-0.324  -0.078-0.067 | 0.103  0.873 |
| mCmC  Sex  Age | -0.081  0.050 | -4.66-4.51  -0.13-0.23 | 0.972  0.574 |
| uCmC  Sex  Age | -0.34  -0.19 | -1.73-1.04  -0.074-0.035 | 0.616  0.472 |
| mCuC  Sex  Age | -0.83  -0.016 | -2.11-0.44  -0.067-0.034 | 0.192  0.508 |
| uCuC  Sex  Age | 0.094  0.063 | -2.16-2.35  -0.026-0.152 | 0.933  0.159 |

Table S4 Multivariate regression analysis on 36 Alu-methylated Intestinal metaplasia patients.

|  | Adjusted β | 95 % CI | *p*-value |
| --- | --- | --- | --- |
| Pattern of Alu methylation | | | |
| mC  Sex  Age | 0.58  0.025 | -0.82-1.97  -0.020-0.070 | 0.407  0.264 |
| mCmC  Sex  Age | 0.70  0.020 | -0.55-1.95  -0.020-0.061 | 0.261  0.303 |
| uCmC  Sex  Age | -0.65  -0.12 | -2.01-0.72  -0.056-0.032 | 0.340  0.592 |
| mCuC  Sex  Age | -0.39  -0.00076 | -2.25-1.47  -0.061-0.059 | 0.676  0.980 |
| uCuC  Sex  Age | 0.044  -0.011 | -2.35-2.44  -0.088-0.066 | 0.970  0.773 |

Table S5 Multivariate regression analysis on 15 Alu-methylated Dysplasia patients.

|  | Adjusted β | 95 % CI | *p*-value |
| --- | --- | --- | --- |
| Pattern of Alu methylation | | | |
| mC  Sex  Age | -3.46  -0.10 | -7.90-0.97  -0.33-0.126 | 0.114  0.345 |
| mCmC  Sex  Age | -10.25  -0.54 | -23.89-3.40  -1.25-0.16 | 0.128  0.120 |
| uCmC  Sex  Age | 1.46  0.060 | -0.55-3.47  -0.044-0.16 | 0.139  0.229 |
| mCuC  Sex  Age | 0.065  0.057 | -2.03-2.16  -0.052-0.17 | 0.947  0.276 |
| uCuC  Sex  Age | 1.93  -0.019 | -1.07-4.94  -0.18-0.14 | 0.186  0.790 |

Table S6 Multivariate regression analysis on 29 Alu-methylated adenocarcinoma patients.

|  | Adjusted β | 95 % CI | *p*-value |
| --- | --- | --- | --- |
| Pattern of Alu methylation | | | |
| mC  Sex  Age | -0.83  0.028 | -2.36-0.70  -0.019-0.075 | 0.274 0.230 |
| mCmC  Sex  Age | -4.09  0.011 | -8.16-0.022  -0.11-0.14 | 0.049  0.857 |
| uCmC  Sex  Age | 1.83  0.063 | -0.26-3.91  -0.00084-0.13 | 0.083  0.053 |
| mCuC  Sex  Age | 1.52  -0.040 | -0.88-3.92  -0.11-0.035 | 0.205  0.287 |
| uCuC  Sex  Age | -1.30  -0.060 | -2.70-0.096  -0.10-0.017 | 0.067  0.008 |
